# Inhibitors of human histone deacetylase with potent activity against the African trypanosome *Trypanosoma brucei*

**DOI:** 10.1016/j.bmcl.2012.01.072

**Published:** 2012-03-01

**Authors:** John M. Kelly, Martin C. Taylor, David Horn, Einars Loza, Ivars Kalvinsh, Fredrik Björkling

**Affiliations:** a Department of Infectious and Tropical Diseases, London School of Hygiene and Tropical Medicine, Keppel Street, London WC1E 7HT, UK; b Latvian Institute of Organic Synthesis, Aizkraukles 21, Riga LV-1006, Latvia; c TopoTarget A/S, Symbion, Fruebjergvej 3, DK-2100 Copenhagen, Denmark; d Department of Molecular Drug Research, Faculty of Health and Medical Sciences, University of Copenhagen, Universitetsparken 2, DK-2100 Copenhagen, Denmark

**Keywords:** HAT, human African trypanosomiasis, VSG, variant surface glycoprotein, HDAC, histone deacetylases, DAC, deacetylases, SAR, Structure–activity relationship, Anti-parasitic activity, *Trypanosoma brucei*, Histone deacetylase inhibitors, Hydroxamic acids

## Abstract

A number of hydroxamic acid derivatives which inhibit human histone deacetylases were investigated for efficacy against cultured bloodstream form *Trypanosoma brucei.* Three out of the four classes tested displayed significant activity. The majority of compounds blocked parasite growth in the submicromolar range. The most potent was a member of the sulphonepiperazine series with an IC_50_ of 34 nM. These results identify lead compounds with potential for the development of a novel class of trypanocidal agent.

## Graphical abstract

**Figure:**
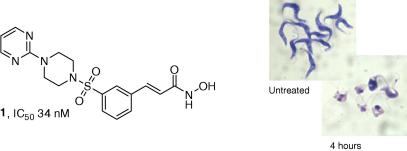

Tsetse fly transmitted protozoa of the *Trypanosoma brucei* species complex are the causative agents of human African trypanosomiasis (HAT). There are currently around 30,000 HAT cases annually, although during epidemics, such as in the late 1990s, this level can increase by more than 10-fold.^1–3^ In domesticated animals these parasites also cause nagana, a disease which has a major impact on agricultural output throughout sub-Saharan Africa. *T. brucei* is an extracellular parasite that avoids immune destruction by a complex process of antigenic variation. This is mediated by periodic switching of the single variant surface glycoprotein (VSG) that covers the parasite cell surface to another antigenically distinct type, encoded by the large repertoire of *VSG* genes.^4^ As a consequence, vaccines are not a realistic option. Drug development is therefore of major importance, and is a WHO priority. Existing therapies for HAT are unsatisfactory for reasons that include severe toxic side effects, increasing resistance, the need for hospitalisation during administration and cost.^5,6^ In the absence of treatment, late stage disease is almost invariably fatal.^5^

Histone modifications play a central role in transcription, chromatin assembly, replication, DNA repair and other regulatory processes central to chromosome biology. For example, coupled acetylation/deacetylation reactions, carried out by histone acetyltransferases and histone deacetylases (HDAC), respectively, are widespread within eukaryotes and act as regulators of numerous cellular events.^7,8^ Aberrant HDAC activity has been associated with a number of different diseases and enzyme inhibitors have broad therapeutic potential.^9,10^ This is particularly the case with cancer, where anti-proliferative effects can result from induction of cell cycle arrest and apoptosis.^11^ In *T. brucei*, there are four putative non-sirtuin HDAC isoforms, of which two (DAC1 and DAC3) are essential in bloodstream form parasites^12^ and play distinct roles in the telomeric silencing of non-expressed *VSG* genes.^13^ Evidence also suggests a role for histone acetylation in epigenetic regulation of RNA polymerase II polycistronic transcription.^14^ Previous studies have demonstrated that inhibitors of HDAC can have significant anti-malarial and anti-leishmanial properties,^15–17^ in addition to activity against a range of other parasites. For instance, the HDAC inhibitor Trichostatin A suppresses growth of bloodstream form *T. brucei*,^18^ and is an inhibitor of DAC1 and DAC3 activity.^13^ Here, we report the efficient growth inhibition of cultured bloodstream form *T. brucei* using a series of inhibitors of human HDACs.

A representative set of HDAC inhibitors was selected by screening our large compound library. These compounds were originally prepared as part of a programme to identify HDAC inhibitors with anti-cancer properties. This resulted in the discovery of the drug candidate Belinostat®, currently in phase III clinical trials. The selected compounds were all hydroxamic acid derivatives, a common structural feature of HDAC inhibitors due to the high affinity of this group for the Zn(II) ion in the metalloenzyme. Four hydroxamic acid compound subclasses, belonging to separate patent series,^19–22^ the sulphoneamides, sulphonepiperazines, long chain amides and a heterocyclic series, were chosen for screening against trypanosomes.

Compounds **1**–**8**, **10**–**15** and **17**–**19,** which are hydroxamic acids with a sulphoneamide or sulphonepiperazine linker in the molecule, were prepared according to previously described procedures.^19,20^ Briefly, they were synthesized from the corresponding sulphonyl chloride and amide or piperazine, followed by the transformation of the methyl ester to the desired hydroxamic acid (Scheme 1, Table 1). Hydroxamic acid derivatives with a long chain amide linker, compounds **21**–**26**, were synthesized by amide coupling between the methyl 6-aminohexanoate and the respective acid, followed by transformation of the ester to the corresponding hydroxamic acid (Scheme 2, Table 1).^21,23^ Finally, the heterocyclic hydroxamic acids **9**, **16**, **20**, were similarly prepared using published procedures.^22^

The compounds from the different structural subclasses were all active as HDAC inhibitors, measured in an assay of enzymes in HeLa cell lysates.^23,24^ HeLa cells express multiple HDACs, including HDAC 1, 2, 3, 4, and 8.^25–27^ The compounds showed broad selectivity, with submicromolar activities (Table 1). To further characterise the compounds with respect to HDAC selectivity, we determined their inhibitory properties against recombinant expressed human HDACs (rhHDAC 1–4, 6–9).^28^ These assays showed that the compounds had a broad and potent inhibitory activity towards these enzymes which belong to the different HDAC subclasses (class I, HDAC 1, 2, 3, 8; class IIA, HDAC 4, 7, 9; class IIB, HDAC 6) (Table 2).

When we investigated the potency of the inhibitors against cultured bloodstream form *Trypanosoma brucei* (strain 427),^29,30^ we found that three out of the four subclasses had significant activity, with growth inhibition occurring in the submicromolar range (Table 1, Fig. 1). The most active compounds were the sulphonepiperazines, particularly those with an aromatic substitution attached to the piperazine moiety. Compounds **1**–**4**, **6**, **7**, and **10**–**12** are examples of the closely related analogues which exhibit similar levels of potent activity (IC_50_ 0.034–0.20 μM, Table 1). Treatment of parasites with micromolar levels of these inhibitors could result in cell death within 4 h (Fig. 1). In this sulphonepiperazine series, the close SAR suggests that further modification of the scaffold could enhance the trypanocidal properties. The sulphoneamide analogues **5**, **8**, **15**, **17**–**19** also displayed significant potency against bloodstream form parasites (IC_50_ 0.14–0.93 μM, Table 1), which showed a clear SAR. Interestingly, although analogues from both classes inhibited HeLa cell HDACs at a range of submicromolar concentrations (IC_50_ 0.010–0.19 μM), there was no obvious correlation with the pattern of activity observed against trypanosomes.

The heterocyclic compounds **9** and **16**, which are close quinoline analogues, displayed similar trypanocidal properties (Table 1). However compound **20**, a benzoxazole, thus structurally distinct from the other two heterocycles was 5- to 10-fold less active. The amide series, which contains a C5 carbon linker (compounds **21**–**26**), did not show activity against *T. brucei* (up to 10 μM, Table 1), despite being very potent inhibitors of HeLa cell HDACs. Comparison of the trypanosome inhibitory properties of compounds **1**–**20**, which have an aromatic group attached close to the hydroxamic acid, with those of the inactive amide series (compounds **21**–**26**), suggests that structural differences between human and *T. brucei* target enzymes may influence the efficiency of inhibitor binding. Importantly, the selectivity implies that the trypanocidal activity results from specific interaction(s), rather than from general metal ion chelating properties. Therefore, the disparity in activity profiles of these compounds against human and parasite cells could be due, at least in part, to their differential abilities to inhibit the corresponding HDACs, or other metalloproteins. Interestingly, the two essential *T. brucei* HDACs (DAC 1 and 3) are divergent, relative to their mammalian counterparts, as are their histone substrates. The major differences lie in the amino and carboxyl extensions beyond the more conserved catalytic core. There is also a large (174 amino acid) insert within the core of the class II enzyme, DAC3.^12^ The structures of these enzymes have yet to be determined. However, HDAC inhibitors typically function through chelation of the active site zinc ion, and the DAC3 insert, as well as the other differences, may have an impact on access to the active site pocket for both substrates and inhibitors. It is notable here that highly specific selective inhibitors have been identified for human HDACs^31,32^ and the differences between the human and *T. brucei* enzymes might also be exploited for the development of trypanosome selective inhibitors.

In conclusion, by screening a library of compounds produced for anti-cancer studies, we have identified leads with potential for treating African trypanosomiasis. These data provide a platform for the definitive identification of the target enzyme(s) within the parasite and the informed design of more potent compounds with optimised pharmacokinetic properties.

## Figures and Tables

**Figure 1:**
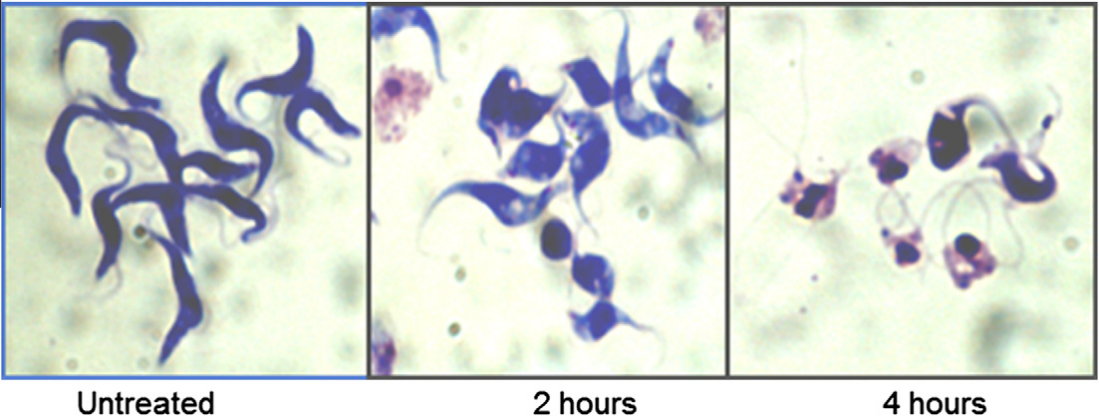
Bloodstream form *T. brucei* bloodstream treated with compound **1** (2 μg/ml). At the time points indicated, parasites were fixed methanol, stained with Giemsa and visualised using a Leica DMRB microscope.

**Scheme 1:**
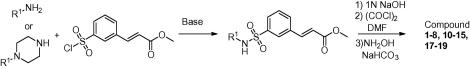
Preparation of hydroxamic acids with a sulphoneamide or sulphonepiperazine linker.

**Scheme 2:**
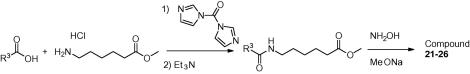
Preparation of hydroxamic acids with a long chain amide linker.

**Table 1:** Prepared HDAC inhibitors and biological activity^23,24,29,30^

**Figure:**
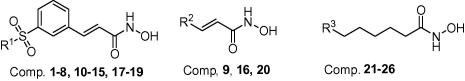

Compound	R^1^	R^2^	R^3^	*Trypanosoma brucei* (BSF)		HDAC (FDL_HELA)
				IC_50_ ± SD (μM)	IC_90_ ± SD (μM)	IC_50_ ± SD (μM)
**1**				0.034 ± 0.002	0.066 ± 0.004	0.084 ± 0.053
**2**				0.064 ± 0.005	0.130 ± 0.005	0.149 ± 0.006
**3**				0.086 ± 0.009	0.201 ± 0.005	0.123 ± 0.076
**4**				0.123 ± 0.006	0.169 ± 0.008	0.187 ± 0.082
**5**				0.137 ± 0.007	0.22 ± 0.007	nd
**6**				0.141 ± 0.009	0.224 ± 0.014	0.191 ± 0.036
**7**				0.154 ± 0.013	0.281 ± 0.019	0.068 ± 0.036
**8**				0.154 ± 0.013	0.306 ± 0.061	0.01 ± 0.002
**9**				0.155 ± 0.018	0.310 ± 0.040	0.028 ± 0.004
**10**				0.156 ± 0.003	0.224 ± 0.003	0.212 ± 0.112
**11**				0.167 ± 0.012	0.228 ± 0.002	0.132 ± 0.103
**12**				0.198 ± 0.005	0.265 ± 0.005	0.022 ± 0.002
**13**				0.198 ± 0.021	0.333 ± 0.028	0.022 ± 0.002
**14**				0.261 ± 0.038	0.485 ± 0.043	0.269 ± 0.087
**15**				0.343 ± 0.017	0.710 ± 0.017	0.02 ± 0.004
**16**				0.353 ± 0.016	0.648 ± 0.020	0.022
**17**				0.368 ± 0.023	0.651 ± 0.083	0.042 ± 0.011
**18**				0.606 ± 0.081	0.956 ± 0.035	0.013 ± 0.005
**19**				0.927 ± 0.057	2.15 ± 0.14	0.023 ± 0.005
**20**				1.54 ± 0.06	2.18 ± 0.18	0.048 ± 0.028
**21**				>10		0.029 ± 0.016
**22**				>10		0.02 ± 0.002
**23**				>10		0.026 ± 0.036
**24**				>10		nd
**25**				>10		0.018 ± 0.006
**26**				>10		0.006 ± 0.003

**Table 2:** Inhibition of rhHDAC isoforms^a^

Compound/rhHDAC	ED_50_(nM ± SD)							
	**1**	**2**	**3**	**4**	**6**	**7**	**8**	**9**
**3**	nd	55 ± 6	2.8 ± 0.4	nd	179 ± 115	100 ± 97	581 ± 334	86 ± 61
**10**	76 ± 6	36 ± 0.7	295 ± 290	870	107 ± 13	nd	955 ± 166	1179
**12**	53 ± 3	310 ± 28	96 ± 46	nd	305 ± 19	nd	328 ± 193	145 ± 151
**15**	44 ± 0.1	221 ± 141	89 ± 32	65 ± 20	35 ± 16	216 ± 133	211 ± 42	109 ± 56
**18**	19 ± 1	105 ± 64	23 ± 2	33 ± 1	36 ± 3	42 ± 22	167 ± 72	26 ± 3
**20**	134 ± 6	554 ± 44	97 ± 0.6	373 ± 73	68 ± 13	286 ± 8	573 ± 30	278 ± 65
**25**	7.3 ± 1.7	7.2 ± 1.0	9.2 ± 1.9	6.6 ± 0.7	14 ± 9	8.4 ± 2.0	3033 ± 226	7.4 ± 4.3

nd = not determined.

a Each compound was assayed in triplicate per plate. EC_50_ values were determined from the average of a minimum of two plates. Results are means ± SD.
